# Increasing temperature alters the within-host competition of viral strains and influences virus genetic variability

**DOI:** 10.1093/ve/veab017

**Published:** 2021-02-23

**Authors:** Cristina Alcaide, Josep Sardanyés, Santiago F Elena, Pedro Gómez

**Affiliations:** 1 Departamento de Biología del Estrés y Patología Vegetal, Centro de Edafología y Biología Aplicada del Segura (CEBAS), CSIC, PO Box 164, 30100 Murcia, Spain; 2 Centre de Recerca Matemàtica (CRM), Edifici C, Campus de Bellaterra, Cerdanyola del Vallès, Barcelona 08193, Spain; 3 Dynamical Systems and Computational Virology Associated Unit Instituto de Biología Integrativa de Sistemas (I2SysBio) - CRM, Edifici C, Campus de Bellaterra, Cerdanyola del Vallès, Barcelona 08193, Spain; 4 I2SysBio, CSIC-Universitat de València, Paterna, 46980 València, Spain; 5 The Santa Fe Institute, Santa Fe, NM 87501, USA

**Keywords:** plant virus, evolutionary ecology, genetic variability, mixed infections, environmental factors

## Abstract

Environmental conditions can affect viral accumulation, virulence and adaptation, which have implications in the disease outcomes and efficiency of control measures. Concurrently, mixed viral infections are relevant in plants, being their epidemiology shaped by within-host virus–virus interactions. However, the extent in which the combined effect of variations in abiotic components of the plant ecological niche and the prevalence of mixed infections affect the evolutionary dynamics of viral populations is not well understood. Here, we explore the interplay between ecological and evolutionary factors during viral infections and show that isolates of two strains of *Pepino mosaic potexvirus* coexisted in tomato plants in a temperature-dependent continuum between neutral and antagonistic interactions. After a long-term infection, the mutational analysis of the evolved viral genomes revealed strain-specific single-nucleotide polymorphisms that were modulated by the interaction between the type of infection and temperature. These results suggest that the temperature is an ecological driver of virus-virus interactions, with an effect on the genetic diversity of individual viruses that are co-infecting an individual host. This research provides insights into the effect that changes in host growth temperatures might have on the evolutionary dynamics of viral populations in mixed infections.

## 1. Introduction

Plant viral diseases cause widespread epidemics in crops and hamper the sustainability of food production, with severe ecological, socio-economic, and political consequences (Vurro, Bonciani, and Vannacci 2010; Lefeuvre et al. 2019). Among many others, notable examples of viral diseases affecting crops are *Tomato yellow leaf curl begomovirus*, which is devastating tomato production worldwide (Moriones and Navas-Castillo 2000), *Tomato spotted wilt orthotospovirus* which re-emerged affecting more than 800 plant species, *Yellow mottle sobemovirus* disease affecting irrigated rice (Fargette et al. 2008), and recently, the cassava mosaic geminiviruses that are threatening cassava production (Legg et al. 2015). The emergence and spread of infectious viral diseases is associated to intrinsic viral and host components, as well as to environmental, agronomical and socioeconomical factors (Anderson et al. 2004; Rojas and Gilbertson 2008; Elena, Fraile, and García-Arenal 2014; González, Butković, and Elena 2019; González, Butković, and Elena 2020; González et al. 2021). While environmental heterogeneity can shape plant–virus infection networks at spatio-temporal scales, which seems to be a key driver of the disease emergence and dynamics (Hily et al. 2016; Rodríguez-Nevado et al. 2018; Valverde et al. 2020), the impact of a fundamental plant niche dimension, the environmental temperature, on virus evolution is unclear. It has been shown experimentally that symptom expression and virus accumulation can be influenced by temperature, with temporal fluctuations during the plant infection process (Obrępalska-Stęplowska et al. 2015; Chung et al. 2016). In fact, changes in temperature affect the host plant–virus interaction (Harrison 1956; Kassanis 1957) and have been correlated with modifications in host gene expression and metabolism changes that lead to variations on plant growth (Went 1953), subsequent symptom severity and within-host virus accumulation (Király et al. 2008; Aguilar et al. 2015; Obrępalska-Stęplowska et al. 2015; Chung et al. 2016; Honjo et al. 2020). In this sense, it is thought that virus accumulation is temperature-dependent, which in fact has been recently shown in natural conditions, revealing that seasonality can affect virus–plant interaction and virus dynamics during persistent infection (Honjo et al. 2020). Thus, it is likely that temperature can impact on viral adaptive evolution by both influencing the genetic variability and the effective population size. However, little is known about how and to what extent temperature can affect viral genetic diversity within-host, and how it could affect the eco-evolutionary dynamics of viral populations.

In parallel, multiple viral infections (i.e. mixed infections) have considerable significance on plant crops (Syller 2012; Syller and Grupa 2016; Tollenaere, Susi and Laine 2016; Alcaide et al. 2020b; Moreno and López-Moya 2020). Viruses can interact when they are infecting the same host. In general, we can divide these interactions in synergistic, neutral and antagonistic, although they can be much more complex (Martín and Elena 2009; Syller 2012; Syller and Grupa 2016; Alcaide et al. 2020b). Thus, viruses can respond to the presence of other viruses co-infecting the same host, where selection processes through new environmental and biotic conditions might shape viral population dynamics compared to single infections (Barraclough 2015; Susi et al. 2015). Hence, within-plant virus–virus interactions could have causal effects on the population structure and epidemiology during an infection (Alcaide et al. 2020b; Sallinen et al. 2020), leading to major consequences on virulence and fitness of the involved viruses (Read and Taylor 2001; Hodgson et al. 2004; de Roode et al. 2005; Tollenaere, Susi and Laine 2016). Within this context, viral co-infections could also affect the mutational load, which for example in the case of the segmented bacteriophage φ6 appears to be purged slower in co-infections than in single infections due to complementation of less-fit genotypes and defective mutants (Froissart et al. 2004). However, similar genetic diversity in protein-coding regions was observed in populations of φ6 at high and low levels of co-infection (Dennehy et al. 2013). This difference can be attributed to the type of mutations assessed. Only deleterious mutations were considered by Froissart et al. (2004), while Dennehy et al. (2013) included beneficial and neutral mutations, with beneficial mutations maintained longer in asexual populations. Moreover, once new variants emerge in a population, viral interference along with the segregation of the variants into different cell tissues could allow coexistence within-host either by a genetic trade-off between competition and colonization (Ojosnegros et al. 2010), or minimizing the strength of selection until reaching a within-host drift-mutation-selection balance (Novella et al. 1995; Saldaña et al. 2003; Froissart et al. 2004; Andino and Domingo 2015). It is therefore that multiple infections may lead to an additional evolutionary advantage of rare variants, increasing genetic diversity, as selection may favor those genotypes that require fewer stabilizing effects to allow coexistence (Lankau 2009).

Together, all these aspects emphasize that the impact of mixed infections on the virus population genetic variability could be largely contingent upon host ecology. This is particularly important since mixed infections may allow diverse and complex interactions within-host, which in turn, could be affected by abiotic factors. Then, it is possible that temperature affect the strength and specificity of virus-virus interactions, influencing differentially the viral fitness, and hence, the evolutionary dynamics of those viruses that are simultaneously infecting the same host. Despite this is an epidemiologically relevant situation, there is currently no information on the potential effect of within-host interactions between strains/genotypes of the same virus in the evolutionary dynamics of viral populations under different environmental conditions.

In this study, we examined the interplay between temperature and mixed infections using two isolates of pepino mosaic virus (PepMV; species *Pepino mosaic potexvirus*, genus *Potexvirus*, family *Alphaflexiviridae*). Both isolates, belonging to the European (EU) and Chilean (CH2) strains (Gómez et al. 2009; Alcaide et al. 2020a), were used as a model of close-related viruses that naturally infect tomato plants. PepMV genome consists of a positive-sense, single-stranded RNA molecule of approximately 6.4 kb in length, containing five open reading frames (ORFs) flanked by two untranslated regions, with a poly(A) tail at the 3′ end of the genomic RNA (gRNA). This virus naturally spreads through mechanical transmission on tomato plants and causes severe economic losses worldwide (Hanssen and Thomma 2010; Gómez-Aix et al. 2019a; Alcaide et al. 2020a). Our previous studies show that the co-occurrence of both EU and CH2 types has been associated for long with recurrent epidemics in Spain (Gómez et al. 2009; Gómez-Aix et al. 2019a; Alcaide et al. 2020a). In addition, despite viral accumulation may depend on the tested virus isolate as well as the analyzed plant host, it has been described that geographically different CH2 isolates had similar decrease in their accumulation levels in the presence of an EU type isolate in two different tomato cultivars, showing that the CH2 type is readily antagonized by the EU type (Gómez et al. 2009; Alcaide et al. 2020a). We hypothesized that temperature could affect this virus–virus interaction, which in turn could shape the within-host genetic diversity of viral populations. To test this hypothesis, we experimentally inoculated tomato plants at 20°C and 30°C with two isolates of PepMV belonging to the EU and CH2 strains, either in single and mixed infections to assess the extent in which temperature and mixed infections affect the within-host accumulation and genetic-variability of PepMV.

## 2. Materials and methods

### 2.1 Plant growth conditions, virus inoculation and sampling

Two infectious PepMV clones, PepMV-Sp13 isolate (EU type) (Aguilar et al. 2002) and PepMV-PS5 isolate (CH2 type) (Gómez et al. 2009; Sempere et al. 2011) were used to agroinfiltrate *Nicotiana benthamiana* Domin plants separately, in order to obtain purified virions of both isolates (Gómez et al. 2009). After 14 days post-inoculation (dpi), viral particles were purified from the homogenized plant tissue, following a series of centrifuges and a PEG precipitation (AbouHaidar, Xu and Hefferon 1998; Gómez et al. 2009). Seeds of tomato plants (*Solanum lycopersicum* L. cv. Money Maker, as a popular cultivated tomato variety) were pre-germinated in a greenhouse with a photoperiod of 16 h light:8 h dark and a temperature between 22 °C and 26 °C until seedling emergence. Then, two sets of 33 seedlings were placed into a greenhouse with controlled temperature conditions at either 20 °C or 30 °C during 2 weeks. Both temperatures were selected based on the knowledge that, in general, the optimum temperature for tomato cultivation is 25 °C, and plant growth rate seems to be affected above and below of this temperature (Shamshiri et al. 2018). After 30 days post germination and for each temperature condition, six tomato plants were mock-inoculated and nine plants per treatment were mechanically inoculated either with EU type, CH2 type or both isolates simultaneously in a proportion 1:1. Virus inoculations were carried out on the third and fourth true leaves by rubbing Carborundum-dusted and a suspension of virions particles in sodium phosphate buffer (30 mM), as previously described (Gómez et al. 2009). During the experiment, PepMV infection was checked by the detection of both type strains from a leaf-sample of each plant collected at 12, 30 and 58 dpi. Total RNA was extracted using Tri-reagent, and 1 µl of RNA was placed on a nylon membrane and fixed with ultraviolet light (CL-1000 Ultraviolet Crosslinker). PepMV infections were detected by dot-blot molecular hybridization using specific RNA probes to specifically detect EU and CH2 types (Gómez-Aix et al. 2019b), following the Roche protocol (RNA labelling and detection kit), and revealing the membrane in a chemiluminescent detector Amersham Imager 600. Additionally, another set of tomato plants was mechanically inoculated with EU or CH2 types in single and mixed infections (five plants per treatment) under similar conditions in order to estimate viral accumulation at early stages of the infection (7 dpi).

### 2.2 Quantitative *in Planta* viral infections

For two different groups of plants, all apical leaves, except the basal inoculated leaves, of each plant within a group were collected to estimate the viral RNA accumulation either at 7 or 60 dpi. Reverse-transcriptase real-time quantitative polymerase chain reaction (RT-qPCR) was performed by using the Power SYBR Green RNA-to-CT 1-Step Kit (Applied Biosystems), including three technical replicates per sample and following the manufacturer’s recommendations. Ten-fold serial dilutions of viral RNA from the disassembled EU and CH2 virions were stocked at −80 °C and used to generate standard curves during the absolute quantification by RT-qPCR. The copy number of PepMV genomes was calculated from the *C_t_* values and using the molecular weight of PepMV genome per ng of total RNA. Note that in mixed infection treatment two RT-qPCR had to be done per sample, one with EU specific primers and another with those of the CH2 (Gómez et al. 2009).

### 2.3 Full-length viral amplification

Plant material was collected at 60 dpi. The top eight leaves of plants were harvested and grinded in a mortar using liquid N_2_. Although, analysis of individual samples may be more reliable, in this case, three pools were made per treatment, with three plants per pool. A sample from each pool was taken and RNA extraction was performed using RNeasy Plant Mini Kit (QIAGEN) and stored at −20 °C until use. One microgram of total RNA was used for retro-transcription (RT) (Expand Reverse Transcriptase, Roche). In the case of mixed infections, RT reactions were performed in duplicate, one of them using a specific primer for the EU strain (EU_rv: 5′-TTTTTTTTTTTTTTTTTCAAAGAAATAATTA-3′) and another using a specific primer for the CH2 strain (CH2_rv: 5′-TTTTTTTTTTTTTAGTAGATTTAGATACTA AG-3’). Then, cDNA was amplified using Phusion High-Fidelity DNA Polymerase (Thermo Fisher Scientific) and specific primers for each isolate (EU_rv and EU_fw: 5′-CGCGGATCCGGAAAACAAAATAAATA AATAAATATAC-3′ for EU and CH2_rv and CH2_fw: 5′-GAAAACAAAACATAACACATAATATCAAAAGTGACC-3′ for CH2). The initial denaturation was done at 98°C for 1 min, continued by 20 cycles of denaturation at 98°C for 10 s, annealing at 48°C for 30 s and extension at 72°C for 3 min and 30 s, followed by a final extension at 72°C for 7 min. Amplification was checked by electrophoresis in 1 per cent agarose gel. Two PCRs were made per sample (duplicates), mixed and purified using GENECLEAN^®^ Turbo Kit (MP Biomedicals).

### 2.4 Next-generation sequencing of full-length viral genomes

After full-length amplification, a total of 26 cDNA fragments were sequenced: six cDNA fragments from EU type single infections (three from plants grown at 20°C and other three from plants grown at 30°C), six from CH2 type single infection (three for each temperature), twelve from mixed infected plants (three for each isolate and temperature), and also both genomes from the disassembled virions used as starting inoculum were included. Library preparation was performed using the Illumina Nextera XT library preparation kit. Then, libraries were sequenced on the Illumina MiSeq platform, using 250 bp paired-end sequencing reads.

### 2.5 Next-generation sequencing data analysis

The quality of the raw data was determined using FastQC (Andrews 2014), and then trimmed by Trimmomatic (Bolger, Lohse, and Usadel 2014), which allowed us to remove adapters and trimming low quality bases (Phred score > 30). The quality reads were mapped against the reference genome using bowtie2 (Langmead and Salzberg 2012) and alignments were processed by SAMtools (Li et al. 2009). The SNP calling was performed with different variant callers: LoFreq (Wilm et al. 2012), FreeBayes (Garrison and Marth 2012) and SNPGenie (Nelson, Moncla and Hughes 2015). Typically, we considered as true variants all those with a frequency >1 per cent, with a minimum raw depth of 3,000 and neglected those variants that were already present in the samples used as starting inocula. Finally, SNPGenie software was also used for estimation of genetic variability indexes such as nucleotide diversity (*π;* mean number of pairwise nucleotide differences per site across the whole genome) and observed heterozygosity (*H*; mean gene diversity at all polymorphic nucleotide sites in the genome).

### 2.6 Statistical analysis

Exploratory analyses were done with SPSS Statistics version 26 (IBM Corp., Armonk, USA) using generalized linear models (GLM). Viral load (*VL*) data were fitted to a model that incorporates three orthogonal factors, one random nested factor, and one covariate. The orthogonal factors were the type of isolate (*A* ∈ {EU, CH2}), the type of infection (*I* ∈ {single, mixed}) and the experimental temperature (*T* ∈ {20, 30} °C). The five replicate plants (*R* ∈ {1, …, 5}) was nested within the interaction of the three orthogonal factors. Time was used as covariate (*t* ∈ {7, 60} dpi). The full model equation reads:

(1) $${VL}_{\mathrm{ijklm}}\left(t\right)∼\lambda +t+A_{i}+t\times A_{i}+I_{j}+t\times I_{j}+T_{k}+t\times T_{k}+{\left(A\times I\right)}_{ij}+t\times {\left(A\times I\right)}_{ij}+{\left(A\times T\right)}_{ik}+t\times {\left(A\times T\right)}_{ik}+{\left(I\times T\right)}_{jk}+t\times {\left(I\times T\right)}_{jk}+{\left(A\times I\times T\right)}_{\mathrm{ijk}}+t\times {\left(A\times I\times T\right)}_{\mathrm{ijk}}+R{\left(A\times I\times T\right)}_{\mathrm{ijkl}}+t\times R{\left(A\times I\times T\right)}_{\mathrm{ijkl}}+\varepsilon_{\mathrm{ijklm}}\left(t\right),$$

where *λ* corresponds to the grand mean value of *VL* and *ε* represents the sampling error. A Gamma distribution with a log-link function was assumed based on its lowest BIC = 5347.799 (Akaike’s weight *W *=* *1) compared to a Tweedie distribution with log-link (BIC = 5415.938, *W *=* *1.599 × 10^−15^) or identity-link functions (BIC = 5584.627, *W *=* *3.745 × 10^−52^) and with a Normal distribution with an identity-link function (BIC = 5979.395, *W *=* *7.090 × 10^−138^).

The count of SNPs (*SC*) per sampled population 60 dpi were fitted to a model that incorporates *A*, *I* and *T* as orthogonal factors and uses three replicate plants to estimate the error. In this case, the model equation reads

(2) $${SC}_{\mathrm{ijkl}}∼\sigma +A_{i}+I_{j}+T_{k}+{\left(A\times I\right)}_{ij}+{\left(A\times T\right)}_{ik}+{\left(I\times T\right)}_{jk}+{\left(A\times I\times T\right)}_{\mathrm{ijk}}+\xi_{\mathrm{ijkl}},$$

where *σ* corresponds to the grand mean value of *SC* and *ξ* represents the sampling error. A Poisson distribution with log-link function was assumed based on its lowest BIC = 165.554 (*W *=* *0.793) compared to a Tweedie distribution with log-link (BIC = 169.620, *W *=* *0.104) or identity-link functions (BIC = 169.620, *W *=* *0.104) and a negative Binomial with log-link function (BIC = 218.456, *W *=* *2.579 × 10^−12^).

The significance of each term in the models was evaluated using a likelihood-ratio test (LRT) which asymptotically follows a *χ*^2^ distribution. In addition, the magnitude of the effect associated with each term was evaluated using the $\eta_{P}^{2}$ statistic which measures the proportion of total variability in the trait attributable to each factor in the model. Conventionally, values of $\eta_{P}^{2}$ ≥ 0.15 are considered as large effects (Cohen 1988). Goodness-of-fit of the two fitted GLM models was evaluated using McFadden’s pseudo *R*^2^ (McFadden 1974).

### 2.7 Data availability

All sequencing information and data that support the findings of this study have been deposited in the NCBI GenBank with the BioProject code PRJNA639566, under accession numbers SRR12017758 - SRR12017783. Data obtained from all the analyses performed in this work are available from the corresponding author upon reasonable request.

## 3. Results

### 3.1 Identifying significant drivers of PepMV accumulation

**Figure 1.**
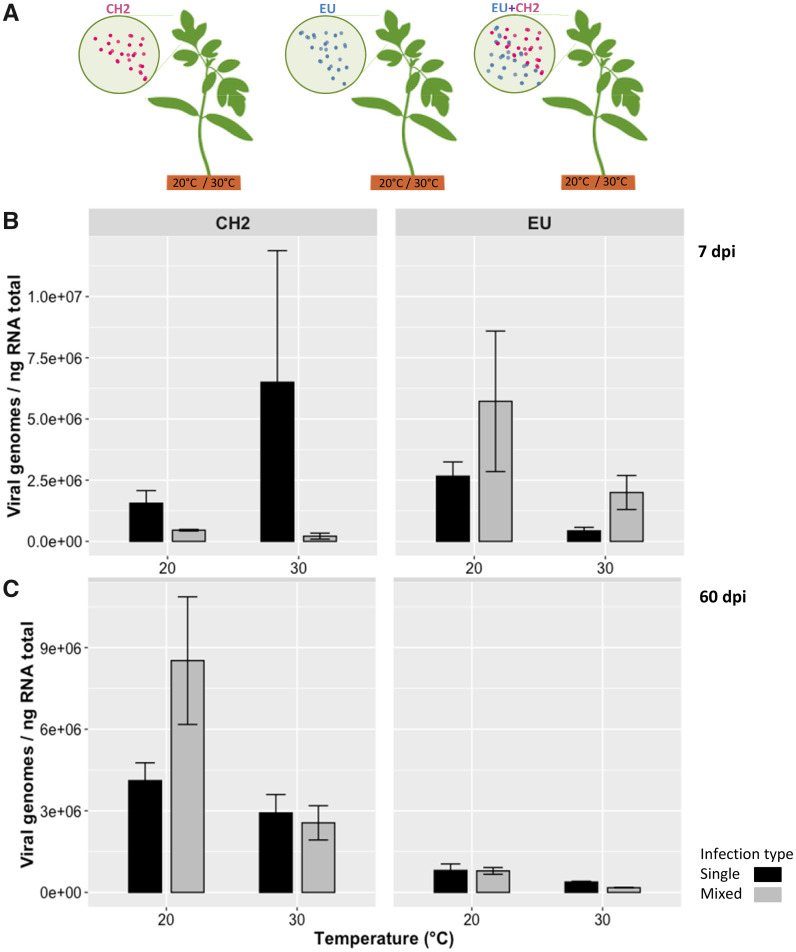
(A) Predicted effect of mixed viral infections on the fitness and genetic diversity of viral populations within an individual host. (B and C) Barplots showing the viral load (viral genomes/ng RNA total) of each PepMV (CH2 and EU) strain in tomato plants grown at 20 °C and 30 °C under single (black) and mixed infection (grey) condition. Viral accumulation was inferred by absolute quantification using RT-qPCR after 7 dpi (B) and 60 dpi (C).

To study whether the PepMV isolates could respond differentially during the infection process, we first investigate how temperature (*T*), type of infection (*I*) and genetic differences among PepMV isolates (*A*) affected gRNA accumulation in tomato plants at 7 and 60 dpi (Fig. 1A). The viral load (VL) data were fitted to the Equation (1). Table 1 shows the statistical summary of the corresponding GLM analysis. Only the interaction term *A *×* T* was not significant. However, a number of terms in Equation (1) (Table 1, bold) had low statistical power (1 − *β *<  0.800), thus wrongly failing to reject the null hypothesis (type II error), and the magnitude of the effect classified as small according to the $\eta_{P}^{2}$ < 0.15 criterion. Thus, hereafter we comment on highly significant and relevant terms. First, regarding the main factors, only the *A* and *T* were relevant by themselves to explain the observed variability in *VL* (Table 1, Fig. 1B and C). Overall, CH2 accumulates to higher concentrations than EU. Remarkably, the interaction between the three main factors and *t* was also highly significant, indicating that the observed effects changed with time. While *VL* was 3.1-fold larger for EU 7 dpi than for EU 60 dpi and this pattern holds in mixed infections, it was 8.0-fold larger for CH2 60 dpi than for CH2 7 dpi. *VL* was, on average, larger in single than in mixed infections, but the magnitude of this difference slightly decreased along the progress of infection (1.8-fold at 7 dpi, and 1.3-fold at 60 dpi). At 7 dpi, infections at 20 °C were 3.3 times more productive than at 30 °C, slightly less (2.7-fold) at 60 dpi.

**Table 1.** Results of the GLM fitting of the viral load data to Equation (1).

Source of variation	LRT^a^	df	*P*	$\eta_{P}^{2}$	1 – *β*^b^
Full model	698,703	61	<0.001		
*λ*	5399.943	1	<0.001	0.993	1.000
*t*	128.340	1	<0.001	0.483	1.000
*A*	298.137	1	<0.001	0.455	0.889
*t *×* A*	417.126	1	<0.001	0.913	1.000
*I*	**129.466**	**1**	**<0.001**	**0.165**	**0.342**
*t *×* I*	67.675	1	<0.001	0.352	1.000
*T*	294.315	1	<0.001	0.410	0.826
*t *×* T*	70.774	1	<0.001	0.352	1.000
*A *×* I*	280.915	1	<0.001	0.416	0.836
*t *×* A*×*I*	260.109	1	<0.001	0.750	1.000
*A *×* T*	**3.630**	**1**	**0.057**	**0.003**	**0.054**
*t *×* A*×*T*	**12.626**	**1**	**<0.001**	**0.044**	**0.649**
*I *×* T*	**71.393**	**1**	**<0.001**	**0.071**	**0.161**
*t *×* I*×*T*	**8.810**	**1**	**0.003**	**0.025**	**0.422**
*A *×* I*×*T*	**146.124**	**1**	**<0.001**	**0.176**	**0.363**
*t *×* A*×*I *×* T*	81.264	1	<0.001	0.391	1.000
*R*(*A *×* I*×*T*)	511.786	14	<0.001	0.857	1.000
*t *×* R*(*A *×* I*×*T*)	257.093	14	<0.001	0.779	1.000

MacFadden’s pseudo *R*^2^ = 0.871.

a Likelihood ratio test, asymptotically distributed as a *χ*^2^.

b Power of the test. Shadowed terms are not further considered because their low statistical power (1 − *β *<  0.800) or small magnitude effect ($\eta_{P}^{2}$ < 0.150).

Second, the only relevant pairwise interaction was *A *×* I* (Table 1, Fig. 1B and C): EU accumulated 1.6-fold more in mixed than in single infections, whereas for CH2, the situation was the reverse: it accumulated 4.2-fold more in single than in mixed infections. Indeed, these differences strongly depend on *t*: while EU productivity in mixed infections was 2.6-fold higher 7 dpi, it was only 1.9-fold higher 60 dpi; in the case of CH2, it accumulated 8.9-fold more in single infections 7 dpi but 1.2-times more in mixed infections 60 dpi.

Third, the tree-ways interaction between the main factors was not significant *per se*, but only throughout a dependence with the duration of infection (Table 1). In the case of EU, *VL* was larger for mixed infections at 7 dpi (the precise magnitude depending on *T*), while at 60 dpi it was larger for single infections (again, the precise magnitude depending on *T*). The opposite pattern was observed for CH2: VL was larger in single infections 7 dpi and slightly larger in mixed infections after 60 dpi at 20 °C, yet the reverse was found at 30 °C.

### 3.2 Mutation occurrence from full-length genome sequencing

**Figure 2.**
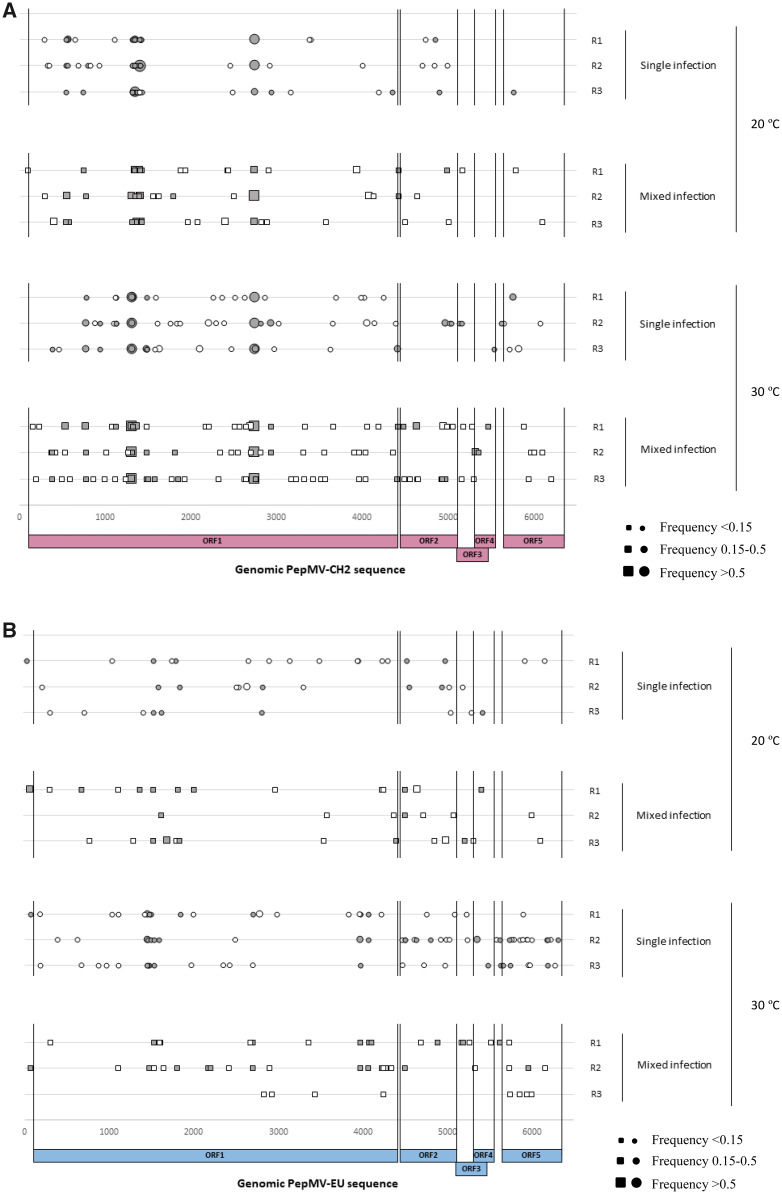
Occurrence and distribution of sites exhibiting mutations in the full-length genome of both PepMV isolate populations; (A) PepMV-PS5 (CH2 type) and (B) PepMV-Sp13 (EU type). The SNPs are marked as synonymous mutations in single (○) and mixed (□) infections, and non-synonymous mutations in single (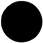) and mixed (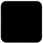) infections for each replicate (*n *=* *3) under 20 °C and 30 °C conditions. The size of the marker indicates the frequency of each SNP.

To examine the influence of differences in accumulation and competition between CH2 and EU on the viral genetic variability within-populations, we first identified a genomic region at the 3′ end of the viral RNA that allows to distinguish both PepMV types and to amplify full-length genomes from mixed infected samples. This allowed us to obtain whole-genome sequences of each PepMV populations after 60 dpi, as well as in the inocula. After cleaning and trimming the raw NGS data, we obtained between 809,756 and 1,777,280 reads per sample, resulting in a theoretical average coverage ranging from 31,750× to 69,687×. These reads were further filtered out by quality, and using the initial inocula as reference, we identified SNPs (and evaluated their frequency in the samples) that appear during the infection experiments. After excluding SNPs present in the inocula and considering variants with a frequency >0.01, including those variants found in the overlapping region between TGB2 and TGB3 genes as double variants because of the potential effect in both proteins, we end up with a total of 519 SNPs (Supplementary Table S1, Figs. 2A and B). No indels or recombination events were observed. Attending to the frequency of the new mutations, the vast majority were ranging from 0.01 to 0.1, with only 3.28 per cent of the total SNPs displaying frequencies > 0.5 (Supplementary Table S1). Note that all these high-frequency mutations were specific to the CH2 populations.

**Figure 3.**
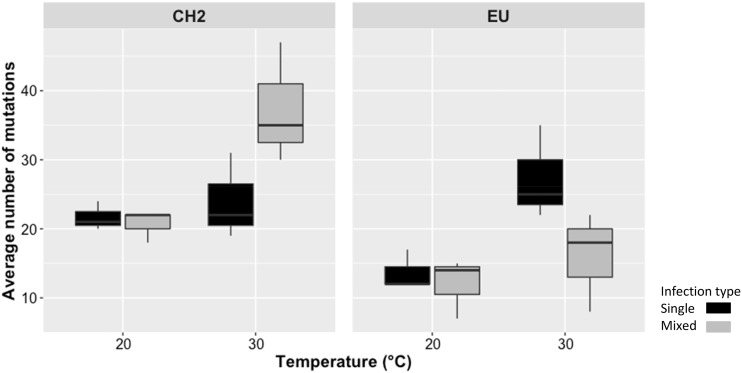
Boxplot displaying the average number of SNPs for each PepMV-CH2 and -EU population in single (black) and mixed infections (grey) at 20 °C and 30 °C of plant growth temperature. The horizontal line shows the median value, and whiskers show the minimum and maximum values.

SNP counts (*SC*) of PepMV within-host populations are summarized in Fig. 3. These data were fitted to the model described by Equation (2) and the results of the analyses are shown in Table 2. Overall, and focusing first on the main effects, highly significant differences of large magnitude existed between the average number of mutations fixed by the CH2 and EU within-host populations. CH2 populations accumulated 1.57-fold more mutations than the EU populations (Fig. 3). No differences existed among single and mixed infections (*I* term in Table 2) in terms of the number of mutations accumulated. The average number of mutations per within-host population also significantly increased 1.57-fold with temperature (*T* term in Table 2 and Fig. 3). The higher-order terms *A *×* I* and *A *×* I*×*T* in Equation (2) were also significant (*P *≤* *0.011) and their effects could be considered of large magnitude ($\eta_{P}^{2}$ ≥ 0.299), suggesting that the observed effect associated with the viral genotype indeed would depend on its interaction with the type of infection and temperature in a nonlinear way. However, given that the power of the two tests was 1 − *β *<  0.8, this conclusion should be taken carefully (Table 2). Furthermore, genetic diversity of CH2 and EU populations was evaluated by using heterozygosity (*H*) and nucleotide diversity (π) estimators (Table 3). We found that both, *H* and π, were higher in the CH2 than EU populations, with averaged *H* values higher at 20°C in mixed infections. However, averaged π values were higher at 30 °C in mixed infections of the CH2 populations that, in turn, was consistent with significant increases of the numbers of synonynous than non-synonymous mutations (Table 3). Hence, conservatively, we can conclude that populations of both PepMV types differed in their mutational load in an infection- and temperature-dependent manner.

**Table 2.** Results of the GLM fitting of the mutation count data to Equation (2).

Source of variation	LRT^a^	Df	*P*	$\eta_{P}^{2}$	1 – *β*^b^
Full model	67.633	7	<0.001		
*σ*	1416.585	1	<0.001	0.955	1.000
*A*	23.662	1	<0.001	0.478	0.949
*I*	0.403	1	0.526	0.000	0.050
*T*	23.155	1	<0.001	0.514	0.971
*A *×* I*	6.993	1	0.008	0.299	0.690
*A *×* T*	0.991	1	0.320	0.000	0.050
*I *×* T*	0.019	1	0.890	0.013	0.072
*A *×* I*×*T*	6.457	1	0.011	0.311	0.713

MacFadden’s pseudo *R*^2^ = 0.952.

a Likelihood ratio test, asymptotically distributed as a *χ*^2^.

b Power of the test. Shadowed terms are not further considered because their low statistical power (1 − *β *<  0.800) or small magnitude effect ($\eta_{P}^{2}$ <0.150).

**Table 3.** Average number of SNPs and estimation of genetic variability indexes (*π*, nucleotide diversity and *H*, observed heterozygosity) for each viral strain population grouped by the temperature and type of infection.

PepMV strain	Temperature (°C)	Type of infection	syn	nsyn	nc	Total	*π* (×10^−4^)	SD (*π*) (×10^−4^)	*H*	SD (*H*)
CH2	20	Single	21	44	0	65	4.584	0.240	0.090	0.008
		Mixed	26	33	2	61	5.400	0.408	0.107	0.016
	30	Single	37	33	2	72	6.591	2.399	0.079	0.043
		Mixed	71	35	8	114	6.910	1.335	0.057	0.023
**EU**	20	Single	25	13	1	39	3.549	0.758	0.049	0.019
		Mixed	20	15	1	36	4.264	0.555	0.071	0.018
	30	Single	50	31	3	84	5.101	1.191	0.065	0.013
		Mixed	29	17	2	48	3.345	0.562	0.043	0.005

Mutations are classified according to the type of change [synonymous (syn) and non-synonymous (nsyn), as well as non-coding variants (nc)].

## 4. Discussion

The interactions between viruses and their host plants are expected to change by altering temperature (Kassanis 1957; Obrępalska-Stęplowska et al. 2015; Honjo et al. 2020). In parallel, accumulating evidence shows a high prevalence of mixed viral infections in plants (Syller 2012; Syller and Grupa 2016; Tollenaere, Susi and Laine 2016; Alcaide et al. 2020b; Moreno and López-Moya 2020). In this work, we have explored the combined effect of a rise in temperature and mixed viral infections. We have connected both, abiotic and biotic factors, and more explicitly, we have sought to explore the relationship between temperature and evolutionary dynamics of viral populations in single and mixed infections. In our studies, different PepMV-CH2 isolates experienced a fitness cost in the presence of an EU-type isolate, suggesting that an asymmetrical antagonistic interaction between these two strains provide a partial explanation for the maintenance of the genetic structure of the PepMV populations in tomato (Gómez et al. 2009; Alcaide et al. 2020a). Both strains have been co-occurring in mixed infections and their long-term co-circulation may have contributed to the evolutionary dynamics of PepMV populations (Alcaide et al. 2020a). However, environmental factors may also affect the virus genetic diversity, and sequencing data from several field isolates hardly inform about the link between environmental factors and virus evolution during epidemic processes. In our experiments, where temperature was controlled and PepMV infections checked at different time-points, we found that the magnitude and sign of the interactions between both isolates appeared to vary according to time and temperature. In the early stage of the infection, 7 dpi, EU is facilitated by CH2 in mixed infections, whilst CH2 is strongly antagonized by EU at both temperatures. However, at the end of the experiment, CH2 showed a higher viral load than EU in single and mixed infections (Fig. 1), and either CH2 or EU are facilitated at 20 °C. Considering these results, along with the efficient PepMV mechanical transmission in the field, it is possible that the observed maintenance of the EU type on tomato crops may be mainly due to its dispersion at early states of the plant growth. Whereas, it seems not to be the case in the later stages. In this sense, a better understanding of the population dynamics at different stages of the plant growth could provide valuable information about the causes of establishment and displacement of viral strains.

Increasing temperature may influence the accumulation of individual viruses co-infecting the same host plant and affect their potential viral interactions. This temperature effect on virus accumulation can be likely due to the potential alterations on the plant physiological and biological traits (Went 1953). For instance, changes on viral titers of *Potato potyvirus* Y^O^ and *Potato potyvirus A* were temperature-dependent, since at high temperatures (25 °C–30 °C), they were able to accumulate in the early stages of the infection but disappeared over time (Chung et al. 2016). In the case of *Turnip mosaic potyvirus* infecting *Brassica campestris*, viral accumulation was lower at elevated temperatures (33 °C) as well as an inhibition of symptoms was observed, which can be explained by an increase of siRNAs involved in the plant silencing pathway (Szittya et al. 2003; Chung et al. 2015). However, temperature can also reduce plant resistance against different pathogens; for example the hypersensitive response induced by different *R* genes against *Tobacco mosaic tobamovirus* (TMV) and *Potato potexvirus X* was reduced by an increase of the temperature, although no determinants of this temperature sensitivity in the plant defence response were identified (Wang et al. 2009). Another study using TMV showed that the stimulated activity of antioxidant enzymes at elevated temperatures (above 28 °C) was associated with the suppression of TMV-induced HR-type necrosis and could have a role in the suppression of the resistance at high temperatures (Király et al. 2008).

We found that virus–virus interactions varied in a temporal scale in a temperature-dependent manner, which had an effect on the nucleotide variation of both viral isolates, depending on the type of infection. Compared to the single infection, the genetic variation of the CH2 population increased in the mixed infections at 30 °C, while the opposite pattern was found for the EU population (Fig. 3). However, at 20 °C, no differences were found in genetic variability between single and mixed infections in both virus populations. Despite the observed differences between 20 °C and 30 °C tell us nothing about the shape of the relationship between evolution rate and temperature (i.e. whether it is monotonously increasing or has an optimum or a minimum at some intermediate value), the conclusion that increasing temperature could indeed modify the viruses’ evolution rates is still robust. As a summary, temperature likely has an impact on the origin and maintenance of the PepMV genetic diversity throughout a relaxation of competitive interactions in mixed infections. In this sense, we speculate that plant phenotypic plasticity and resilience to temperature changes may be playing an important role, suggesting that this framework could be extended to investigate how the tolerance of plants to warmer temperatures affects the viral genetic variability and competitive interactions in mixed infections. Further research can help in anticipating the impact of plant breeding programs for thermotolerance and how new plant cultivars will drive the eco-evolutionary dynamics of viral populations in plant crops.

Most of the information on virus evolution relies on single infections. However, mixed infections are highly prevalent in nature, thus limiting the value and generality of qualitative and, to a much lesser extent, quantitative inferences about emerging viral diseases are gathered from single infections. Viral occurrence and prevalence in plant crops seems to be largely explained by either neutral, synergistic or antagonistic interactions among co-infecting viral species (Martín and Elena 2009; Syller 2012; Syller and Grupa 2016; Alcaide et al. 2020b; Moreno and López-Moya 2020). However, whether these interactions show some degree of genetic plasticity in response to environmental stresses has not been tested so far. This lack of information results from the hardness to distinguish among genetically similar strains of the same virus. This particular bias happened with both CH2 and EU strains. Fortunately, we could solve this problem by focusing into a genomic region at the 3′ end of the viral RNA genome that allowed to obtain the full-length genomes from mixed infections, capturing the nucleotide temporal variation of two related strains. Pending a more reliable approach that may explore genetic variability in mixed infections without ruling out variants with very low frequency within populations, our results can be summarized as follow. First, no recombinant variants were found in the mixed infections experiments, neither in the previous field tomato surveys since 2005 (Alcaide et al. 2020a). This suggests that inter-strain recombinants seem to be deleterious in the virus populations. Second, SNPs were the only mechanism contributing to the genetic variability within PepMV populations after long-term infections. Third, the type of infection (single and mixed) did not influence the average population mutational load at 20°C. The last observation is in accordance with previous studies where the viral population diversity did not differ between mixed and single infections (Dennehy et al. 2013; Ali and Roossinck 2017). However, the situation changed at the higher temperature regardless viral accumulation: both strains displayed higher genetic variation and greater differences in accumulation at higher temperatures (Fig. 2 and Fig. 3). In particular, the CH2 populations displayed a larger number of SNPs in mixed than in single infections at higher temperature, while the opposite seems to be the case for the EU population. Importantly, a rise in temperature in mixed infections resulted in a higher occurrence of synonymous than non-synonymous substitutions in the CH2 population. However, in the EU population and under the same conditions, it was observed a reduction of mutations, although the ratio between both type of substitutions remained at similar levels to those in single infections. This suggests that most of mutations are deleterious, and there is limited understanding of the long-term impact of those mutations *per se*. For instance, analyses of RNA virus species from *in vitro* and natural populations have found that genetic variation mostly comprises transient deleterious mutations that are purged by purifying selection (Elena and Moya 1999; Pybus et al. 2007; Cuevas et al. 2015). However, these mutations could also interact through positive epistasis with a relative effect on fitness (Lalić and Elena 2012; Hillung, Cuevas and Elena 2015; Kutnjak, Elena and Ravnikar 2017; Minicka et al. 2017) and even do so in a host-dependent manner (Lalić and Elena 2013). Thus, uncovering the relationship between those mutations found in this study and fitness, and whether they can contribute to adaptation under temperature and mixed infection conditions, would be the subject of future investigations.

Theoretical and computational studies have shown the importance of competitive ability and viral fitness trade-off in determining the evolutionary dynamics of the viral populations (Sardanyés, Elena, and Solé 2008; Ojosnegros et al. 2010). We speculate that a trade-off between replication rate and competition could have influenced this different mutational effect. Under the same framework, it has been theoretically demonstrated that the trade-off between replication and competition can affect diversity, with a moderate trade-off promoting diversity (Farahpour et al. 2018). For example, it is likely that the EU population had an increased cost between replication and competition in mixed infections under high temperatures, reducing its genetic variability compared to single infections. Different viral combinations with different fitness responses (Alcaide et al. 2020b and references therein) may modify the outcomes of this eco-evolutionary dynamics, thus emphasizing the need of further research.

In conclusion, we have observed a tight connection between temperature and viral interactions in mixed infections that can result in a driver of viral diversity in plants. Due to the changing PepMV virus–virus interactions over time, a rise in temperature may affect the within-host genetic diversity of viral populations in a strain-dependent manner regardless of the viral accumulation in mixed infections. A noteworthy remark is that pathogens evolve not only within hosts but also between hosts, and thus, the frequency of mixed infections and the success of re-infections in one host must be also considered within the evolutionary context. Finally, assessing the mechanistic basis of selective differences will allow us to understand the importance of abiotic and biotic factors in wider pathosystems.

## Supplementary data

Supplementary data are available at *Virus Evolution* online

## Author Contributions

CA, SFE and PG conceived and designed the study. CA and PG conducted the experiments. CA, JS, SFE and PG analysed data and wrote the manuscript.

## Acknowledgements

We thank M.C. Montesinos for technical assistance and three anonymous reviewers for constructive comments. We acknowledge support of the publication fee by the CSIC Open Access Publication Support Initiative through its Unit of Information Resources for Research (URICI).

## Funding

CA was supported by the Ministry of Economy, Industry and Competitiveness (MINECO, Spain) within a PhD programme grant (FPU16/02569). This work was supported by the Agencia Estatal de Investigación-FEDER grants AGL2014-59556-R and AGL2017-89550-R to PG and PID2019-103998GB-I00 to SFE. JS has been partially funded by the CERCA Programme of the Generalitat de Catalunya, MINECO grant MTM2015-71509-C2-1-R, Agencia Estatal de Investigación grant RTI2018-098322-B-I00, and by a Ramón y Cajal contract (RYC-2017-22243).

**Conflict of interest**: None declared.

## Supplementary Material

veab017_Supplementary_Data
